# Impact of the COVID-19 pandemic on exercise habits among cancer patients

**DOI:** 10.21203/rs.3.rs-704646/v1

**Published:** 2021-09-21

**Authors:** Caroline Himbert, Cassandra A. Hathaway, Bailee Daniels, Karen Salas, Anjelica Ashworth, Biljana Gigic, Tengda Lin, Richard Viskochil, Anne C. Kirchhoff, Douglas Grossman, Jennifer Ose, Jonathan Tward, Courtney Scaife, Jane C. Figueiredo, Adetunji T. Toriola, Anna Beck, David Shibata, Brian D. Gonzalez, Cindy Matsen, Cristina Christenson, Debra S. Ma, Howard Colman, Jason P. Hunt, Kevin B. Jones, Catherine J. Lee, Mikaela Larson, Tracy Onega, Wallace L. Akerley, Christopher I. Li, Martin Schneider, Frank J. Penedo, Erin M. Siegel, Shelley S. Tworoger, Cornelia M. Ulrich, Anita R. Peoples

**Affiliations:** Huntsman Cancer Institute Cancer Hospital: University of Utah Health Huntsman Cancer Institute; Moffitt Cancer Center; Huntsman Cancer Institute Cancer Hospital: University of Utah Health Huntsman Cancer Institute; Huntsman Cancer Institute Cancer Hospital: University of Utah Health Huntsman Cancer Institute; Huntsman Cancer Institute Cancer Hospital: University of Utah Health Huntsman Cancer Institute; Heidelberg University: Ruprecht Karls Universitat Heidelberg; Huntsman Cancer Institute Cancer Hospital: University of Utah Health Huntsman Cancer Institute; Huntsman Cancer Institute Cancer Hospital: University of Utah Health Huntsman Cancer Institute; Huntsman Cancer Institute Cancer Hospital: University of Utah Health Huntsman Cancer Institute; Huntsman Cancer Institute Cancer Hospital: University of Utah Health Huntsman Cancer Institute; Huntsman Cancer Institute Cancer Hospital: University of Utah Health Huntsman Cancer Institute; Huntsman Cancer Institute Cancer Hospital: University of Utah Health Huntsman Cancer Institute; Huntsman Cancer Institute Cancer Hospital: University of Utah Health Huntsman Cancer Institute; Cedars-Sinai Comprehensive Cancer Center: Cedars-Sinai Medical Center Samuel Oschin Comprehensive Cancer Institute; Washington University In St Louis: Washington University in St Louis; Huntsman Cancer Institute Cancer Hospital: University of Utah Health Huntsman Cancer Institute; University of Tennessee Health Science Center Bookstore: The University of Tennessee Health Science Center VolShop Memphis; Moffitt Cancer Center; Huntsman Cancer Institute Cancer Hospital: University of Utah Health Huntsman Cancer Institute; Huntsman Cancer Institute Cancer Hospital: University of Utah Health Huntsman Cancer Institute; Huntsman Cancer Institute Cancer Hospital: University of Utah Health Huntsman Cancer Institute; Huntsman Cancer Institute Cancer Hospital: University of Utah Health Huntsman Cancer Institute; Huntsman Cancer Institute Cancer Hospital: University of Utah Health Huntsman Cancer Institute; Huntsman Cancer Institute Cancer Hospital: University of Utah Health Huntsman Cancer Institute; Huntsman Cancer Institute Cancer Hospital: University of Utah Health Huntsman Cancer Institute; Huntsman Cancer Institute Cancer Hospital: University of Utah Health Huntsman Cancer Institute; Huntsman Cancer Institute Cancer Hospital: University of Utah Health Huntsman Cancer Institute; Huntsman Cancer Institute Cancer Hospital: University of Utah Health Huntsman Cancer Institute; Fred Hutchinson Cancer Research Center; Heidelberg University: Ruprecht Karls Universitat Heidelberg; Miami Cancer Institute; Moffitt Cancer Center; Moffitt Cancer Center; Huntsman Cancer Institute; Huntsman Cancer Institute Cancer Hospital: University of Utah Health Huntsman Cancer Institute

**Keywords:** exercise oncology, covid-19, cancer prevention, cancer epidemiology, cancer survivorship

## Abstract

**Purpose:**

There is limited information on how the COVID-19 pandemic has changed health behaviors among cancer patients. We examined the impact of the pandemic on changes in exercise behaviors and identified characteristics associated with these changes among cancer patients.

**Methods:**

Cancer patients (n = 1,361) completed a survey from August-September 2020 to assess COVID-19 pandemic-related changes in health behaviors and psychosocial factors. Patients were categorized into 3 groups: exercising less, exercising did not change, and exercising more. Patient characteristics were compared by exercise groups.

**Results:**

One-third of the patients reported a decreased amount of regular exercise, while 11% reported exercising more during the pandemic. Patients who exercised less were more likely to be unemployed/retired, undergoing active treatment, and had increased pandemic-related alcohol consumption and psychosocial stressors such as loneliness and financial stress (all *p* < 0.05). In contrast, patients who exercised more were younger, female, full-time employed, did not consume alcohol, and had good health status and more social interactions (all *p* < 0.05). Patients who were living in rural areas and did not experience changes in daily life, were also more likely not to experience changes in exercise habits (all *p* < 0.05).

**Conclusion:**

Our results indicate that a significant proportion of cancer patients experienced changes in exercise habits during the first 6 months of the COVID-19 pandemic. Age, sex, employment status, health status, alcohol consumption, and psychosocial factors were associated with changes in exercise behaviors. Providers should monitor for changes in health behaviors, such as exercise, because of their importance in improving cancer survivorship.

## Introduction

The COVID-19 pandemic has challenged healthcare in many ways by overwhelming healthcare capacities, delaying elective procedures, and overall, disrupting medical care (1–4). Long-term impacts of the pandemic within many healthcare disciplines are expected to emerge over the next years or even decades. Within cancer care and prevention, the pandemic has led to delays or deferrals of cancer-related treatments, screening, and surveillance appointments (5–10). For example, weekly colorectal cancer screenings drastically decreased by 86% in 2020 (6, 10, 11). As a result, an increase in more advanced stage cancer cases is expected to emerge in the upcoming years.

Many behavioral factors including physical activity have been established as risk and prognostic factors of cancer (12–16). Collective evidence shows that increased physical activity levels throughout the cancer care continuum may decrease the risk of disease and extend survival, particularly among breast and colon cancer patients (12–16). The American College of Sports Medicine reports that by following these guidelines mortality rates of high incident cancer types (e.g., breast, colon, and prostate cancer) may be reduced by 40 to 50% (17, 18). Physical activity after cancer diagnosis has further shown to improve overall quality of life, fatigue, sleep, mental health including depression and anxiety, and immune responses (19).

There is some evidence that the pandemic has led many individuals to change their health behaviors including physical activity levels and diet, potentially due to stay-at-home policies and closure of gyms (20–23). Experts are concerned that social isolation and COVID-19 restrictions will lead to physical inactivity among cancer survivors (24). A recent study reported that physical activity levels measured in step counts significantly decreased within the general population since the start of the pandemic (20). Another study in breast cancer survivors showed that 90% of the study population reduced their physical activity levels and increased the amount of sedentary behavior (25). This study in breast cancer patients is the only study to date among individuals with cancer to examine changes in exercise behaviors due to the pandemic. However, their findings were limited by the small sample size (n = 37).

In the present study, we identify risk/protective factors for decreased physical activity among cancer patients and survivors. The results of this study will highlight the importance of promoting beneficial health behaviors, including exercise, among cancer patients and survivors especially during and post pandemic. Further, this work will allow clinicians and researchers to 1) know which subgroup of patients need early intervention to prevent decreased physical activity, and 2) identify modifiable targets for future interventions (e.g., social interactions).

## Methods

### Study design and participant selection

As part of the COVID-19 and Oncology Patient Experience Study (COPES) consortium, n = 1,477 adult cancer patients (≥ 18 years), who had visited Huntsman Cancer Institute (HCI) between 2016–2020 and were enrolled in the Total Cancer Care (TCC) study (NCT01091974), the ColoCare Study (NCT02328677), completed a COVID-19 survey (26–28). The majority of the patients at HCI completed the survey electronically, while some completed it in person or over the phone, between August and September 2020. Response rates ranged from 14–57%. N = 1,361 patients diagnosed with stage I-IV cancers (n = 27 with stage 0 were excluded) and had data available on changes in exercise habits (n = 89 with missing data on exercise habits or who exclusively reported “exercising in a different location than normal” were excluded), were included in the present work. The University of Utah Institutional Review Board (IRB) approved this protocol, and all participants provided written informed consent.

### Measures

#### Outcome:

##### Exercise habits (health behavior):

A change in exercise habits was determined based on the following questions: ‘Since the COVID-19 pandemic began (March 2020), have your exercise habits changed?’ (yes or no)’ and ‘Since the COVID-19 pandemic began (March 2020), in what way have your exercise habits changed? Check all that apply’ (‘I do not exercise regularly’, ‘I am exercising less’, ‘I am exercising more’, ‘I am exercising in a different location than normal’, and ‘other’). Since the survey was administered between August and September 2020, the changes assessed in exercise habits were during the first 6 months of the pandemic (March to August/September 2020). Ninety-two percent of patients (n = 1,361/1,477), who participated in the survey completed the questions on changes in exercise habits. Patients were categorized into three groups: exercise less i.e., patients who responded ‘I do not exercise regularly’ or ‘I am exercising less;’ exercise did not change i.e., patients who responded ‘no’ change in exercise habits; and exercise more i.e., patients who responded ‘I am exercising more.’ Patients who provided free-text responses under ‘other’ were categorized on an individual basis. However, patients who exclusively checked ‘I am exercising in a different location than normal’ were also excluded from the analyses.

#### Exposures:

##### Demographic and clinical characteristics

Sociodemographic (sex, age, race, ethnicity) and clinical characteristics (tumor site, tumor stage) were abstracted from electronic medical records. Participants self-reported their body-mass index (BMI; if self-reported BMI was missing, it was abstracted from medical records), current cancer patient status, COVID-19 infection status, and employment status. A measure of overall health status was adapted from the 12-item Short-Form Health Survey quality-of-life (QoL) measure (29). Zip codes were categorized as urban or rural using the Rural-Urban Commuting Area Codes (RUCA) classification system (30).

##### Other health behaviors

Smoking status was either self-reported (current or change in smoking status) or abstracted from medical records (ever smoker i.e., at least 100 cigarettes in their lifetime). Patients also self-reported their alcohol consumption in the past year as well as change in alcohol consumption since the COVID-19 pandemic started.

###### Psychosocial factors:

Participants reported changes in daily life and financial stress in past month on a Likert scale from 1 (not at all) to 5 (a lot/very much) (31). Changes in social interaction were reported by participants on a scale from 1 (much less social interaction) to 5 (a lot more social interaction), which was classified as participants reporting either: fewer social interactions, no change in social interactions, and more social interactions. A single item “How often have you felt lonely in the last month?” scored on a scale of 1 (never) to 5 (always) was adapted from the NIH Toolbox Loneliness instrument assessing perceptions of loneliness, which is an aspect of social relationships (32). An item from the Perceived Stress Scale was used to assess perceived stress, asking “In the last month, how often have you felt difficulties were piling up so high that you could not overcome them?” and scored on a scale of 1 (never) to 5 (often) (33, 34).

### Statistical Analyses

Descriptive statistics (frequencies and percentages for categorical variables and mean and standard deviations for continuous variables) were performed for all variables of interest. One-way analysis of variance (ANOVA) for continuous variables and χ-square tests for categorical variables were used to determine statistically significant differences (*p* < 0.05) between the three exercise behavior groups (exercise less, exercise did not change, exercise more). *T-tests* for continuous variables and χ-square tests for categorical variables were used to determine statistically significant differences (*p* < 0.05) between the two changes in exercise behavior groups (exercise more and less). Statistical analyses were performed using SAS Software version 9.4.

## Results

### Patient characteristics

Table 1 summarizes the patient characteristics for N = 1,361 cancer patients included in the present analyses. Mean age was 61 (range: 20–92) and 54% were female. The majority of patients had been diagnosed with breast (n = 156, 13%), gastrointestinal (n = 173, 15%), or hematologic cancers (n = 231, 19%). Over half were diagnosed with cancer stage I (37%) and II (25%). The patients had on average a BMI of 28.2 kg/m^2^. Most participants were residents from Utah and 26% lived in rural areas. About one-third of patients [n = 439 (32%)] reported that they exercised less, while 152 (11%) patients exercised more during the pandemic. Over half of the study population (57%) reported to not have changed their exercise habits since the pandemic.

### Changes in exercise habits by demographic and clinical characteristics

Table 2 compares demographic and clinical characteristics by change in exercise habits. Patients who started exercising more during the pandemic were on average 5 years younger (mean age: 55 years) as compared to those who exercised less (mean age: 60 years) or did not change their exercise habits (mean age: 62 years) (*p* < 0.0001). Female patients appeared to be exercising more since the pandemic (64% exercising more vs. 56% exercising less, *p* = 0.08). Male patients were more likely to maintain their exercise behavior (44% exercising less vs. 50% did not change exercise vs. 36% exercising more). Patients who were full-time employed were less likely to exercise less (29%) or change their exercise habits (35%) as compared to exercising more (48%; *p* < 0.0001); while unemployed or retired patients were more likely to exercise less as compared to exercise more (63% vs. 42%; *p* < 0.0001). Patients who were able to maintain their exercise habits were more likely to be living in rural areas as compared to those exercising less or more (32% vs. 21% or 19%; *p* < 0.0001); on the other hand, a higher percentage of urban patients reported changes in their exercise habits i.e., they either exercised less (79%) or more (81%).

Changes in exercise habits due to the pandemic varied by tumor site (*p* = 0.002). Patients diagnosed with lung cancer or hematologic neoplasms were more likely to increase exercise during the pandemic and less likely to maintain or decrease exercise, while patients diagnosed with gastrointestinal cancers and melanoma were more likely to decrease exercise than maintain or increase exercise (*p* < 0.0001). Patients with stage IV cancer appeared to exercise more compared to exercise less or maintain exercise habits (23% vs. 16% or 15%; *p* = 0.01). Patients who were currently undergoing active cancer treatment were more likely to decrease exercise and less likely to increase exercise (30% vs. 21%; *p* = 0.02). Overall, those with excellent or very good health status exercised more (23% and 42%, respectively) in comparison with exercising less (6% and 32%) and no change in exercise habits (13% and 40%). In contrast, 63% of those who reported to exercise less fell into either good, fair, or poor health status categories. No differences were observed between the exercise groups when comparing patients’ race, ethnicity, COVID infection status (i.e., whether they tested positive for COVID since March 2020), or BMI.

### Changes in exercise habits by health behaviors

Table 3 compares health behavior characteristics by changes in exercise habits. Changes in exercise habits did not differ by smoking status (*p* = 0.19). Patients who never consumed alcohol in the past year were more likely to increase their exercise levels (59%) compared to exercising less (51%) and no changes in exercise behaviors (54%, *p* < 0.0001). Conversely, patients with the highest alcohol consumption (3–4 times a week to every day) either exercised less (18%) or did not change their exercise habits (16%) as compared to only 10% of those who exercised more (*p* = 0.001). Change in alcohol consumption due to the pandemic was associated with changes in exercise habits (*p* = 0.03). In particular, those who reported an increase in alcohol consumption due to the pandemic were also more likely to exercise less (7%) as compared to exercising more (4%) or reporting a change in their exercise habits (4%). Further, those who reported no change in alcohol consumption due to the pandemic were also more likely not to experience a change in their exercise habits (92%) as compared to exercising less (86%) or more (87%).

### Changes in exercise behaviors by psychosocial factors

Table 4 compares psychosocial factors by changes in exercise habits. The majority of patients (56%) experienced a moderate amount or a lot of changes in their daily life due to the pandemic. Patients who reported ‘not at all’ or ‘a little bit’ change in daily lives were also more likely to report no changes in their exercise habits (26%) as compared to exercising less (8%) or more (14%; *p* < 0.0001); there were no significant differences seen between exercising less and more groups (*p* = 0.14). Overall, most patients reported less social interactions in the past month (65%). Patients who reported more social interactions were more likely to exercise more as compared to exercising less or maintaining their exercise habits (*p* < 0.0001). Feeling lonely, difficulties piling up, and financial stress in the past month had similar trends by the exercise groups. Patients who ‘never’ felt lonely, ‘never’ had difficulties piling up, or were ‘not at all’ financially stressed were also more likely not to experience changes in their exercise habits during the pandemic as compared to those exercising less or more (*p* < 0.0001). Conversely, patients who regularly experienced loneliness, difficulties piling up, or financial stress also exercised less as compared to patients who exercised more or maintained their exercise habits (*p* < 0.0001). However, loneliness, difficulties piling up, and financial stress were not significantly different between exercising less and more groups (*p* = 0.48, 0.06, and 0.42, respectively).

## Discussion

To the best of our knowledge, this is the first large study to investigate changes in exercise behaviors among cancer patients during the first 6 months of the COVID-19 pandemic as well as characteristics associated with these changes. One-third of our study population reported to have decreased the amount of regular exercise. Our results also indicate that age, employment status, overall health status, alcohol consumption, current patient status, and psychosocial factors were associated with changes in exercise behaviors during the pandemic.

A reduction in exercise is especially concerning in the cancer population given the strong evidence of beneficial effects of physical activity on survivorship (12–16, 19, 35). Physical activity before and after cancer diagnosis has been shown to have substantial health benefits for cancer patients (12–16, 19, 35). Physical activity not only reduces the risk of cancer but can also improve cancer outcomes, including survival and recurrence. (12–16, 19, 35). Further, physical activity enhances overall quality of life and physical function particular among individuals with cancer, and may improve other potential comorbidities including diabetes, cardiovascular, or respiratory diseases (19). Although not explicitly asked in this study, challenges in accessing exercise facilities, being at high risk of having poor outcomes if exposed to COVID-19 infection, or finding motivation to exercise due to stay-at-home policies may have contributed to the substantial decrease in exercise activities observed in this study.

Our study provides further support for prior research before the pandemic highlighting the barriers to exercise and determinants of exercise adherence among cancer survivors (36, 37). Older cancer survivors often engage in less activity due to underlying health conditions, a higher prevalence of overweight and obesity, cancer treatment-related side effects such as fatigue and pain (37). Among cancer patients, adherence to exercise interventions does not appear to be influenced by sex, although the data remains inconclusive (36). However, our results indicate that response to the pandemic with respect to increased physical activity was higher among female patients compared to those whose physical activity decreased. In alignment with previous studies showing that employed individuals spend more time in moderate and vigorous physical activity (38, 39), full-time employed patients in our study population were more likely to increase their activity levels since the start of the pandemic. Greater opportunities to workout while working from home and less time spent commuting may explain this observation. On the contrary, unemployed patients may be retired and older or may have lost job due to the pandemic and, hence, less active. Consistent with prior research,(40, 41) urban patients in our study experienced more changes in their exercise habits due to the pandemic, potentially due to stay-at-home policies, closure of gyms, or more time to workout (42, 43).

Our study indicates that patients diagnosed with lung cancer or hematologic neoplasms were more likely to increase exercise during the pandemic, while patients diagnosed with gastrointestinal cancers and melanoma were more likely to decrease exercise than increase or maintain it. This aligns with studies conducted before the pandemic showing reduced engagement in physical activity among patients with gastrointestinal cancer (44, 45). Melanoma patients are also more likely to avoid outdoor activities to minimize sun exposure (46), which may explain reduced exercise levels due to closure of gyms with outdoor exercise being the only alternative. Our data suggest that patients with stage IV disease tend to exercise more. Exercise may be a mechanism for these patients to cope with the high burden of treatment- and disease-related side effects. Our data also showed that patients who were currently undergoing active cancer treatment were more likely to decrease exercise. This may be confounded since patients who are currently undergoing treatment or recently completed treatment are likely to experience treatment-related side effects, impairments from surgical intervention, and may be more prone to infections due to weakened immune system and may want to avoid outdoor activities.

Patients following other healthy lifestyle behaviors such as never drinking alcohol or decreasing alcohol consumption during the pandemic were more likely to exercise more, which is consistent with previous studies (47). Individuals who fall into an overall ‘healthier’ lifestyle cluster considering smoking, nutrition, alcohol, and physical activity, are generally more active (47). Overall, our data suggest that the pandemic contributed to the factors that have been previously identified as barriers to exercise, resulting in less exercise during the pandemic among a significant proportion of cancer patients and survivors.

Strong evidence supports exercise as a coping mechanism for side effects of cancer, its treatment, and related behavioral health challenges including anxiety, depression, fatigue, and sleep difficulties (48–50). Research also shows that individuals with underlying mental health problems, perceived stress, or financial hardship engage in less physical activity (51–53). The pandemic has caused the majority of individuals to experience increased levels of stress and anxiety as well as financial difficulties due to loss of employment, income, or health insurance (8, 54). In addition, social distancing policies and other restrictions to prevent the spread of the virus have reduced the ability to cope with underlying mental health conditions. A recent study reported that individuals who remained active or increased their activity levels during the pandemic have done so to maintain their mental health (55). In contrast, mental health conditions associated with the pandemic have emerged as barriers to exercise (55). Our results confirm these findings within the cancer population. In particular, patients who felt lonely, had less social interactions, were financially stressed, and experienced perceived stress in terms of difficulties piling up were more likely to exercise less and less likely to engage in more exercise. These results elucidate the need to screen for mental health challenges faced by cancer survivors during the pandemic and promote psychosocial programs that address unique needs and challenges within this population.

This study has several strengths and limitations. Exercise habits were self-reported and may be subject to recall bias. However, given the timeliness of the pandemic, the conscious perception of changes in life experienced by many, and prospective design of the study, we assume that patients can more easily recall any behavioral changes. Survey responses may have been biased by the type of survey (in person, phone, electronically). The majority of the study population was White, non-Hispanic/Latino, and from Utah. Thus, the generalizability of our results may be limited and not applicable to those with different racial and ethnic backgrounds, or those from other states who may have had different COVID-19 state-wide policies. However, our study population included a significant proportion from rural residents (26%) making the results more applicable for other states with similar urban-rural proportions. The response rate was moderate and may have introduced selection bias.

The pandemic has impacted everyone’s lives in many ways including changes in health behaviors. Health behaviors such as being physically active are even more important for immune compromised populations like cancer survivors. Our results indicate that many cancer patients and survivors reduced their exercise during the first 6 months of the pandemic. This was more common among patients who had poor health, disruptions to daily life, reduced social interactions, and increased levels of psychosocial stressors such as loneliness and financial stress. This study identifies risk factors for changes to physical activity among cancer patients and survivors as well as highlights targets for future interventions to prevent reduced physical activity and promote increased exercise.

## Figures and Tables

**Table 1:** Study population characteristics (n = 1,361).

Characteristics	N (%)*
Age, **mean ± SD (range)**	61 ± 14 (20–92)
Sex	
Male	637 (47)
Female	724 (53)
Race	
White	1,298 (97)
Asian	12 (1)
American Indian or Alaska Native	11 (1)
Other	12 (1)
Ethnicity	
Hispanic/Latino	49 (4)
Non-Hispanic/Latino	1,213 (96)
Body Mass Index (BMI) (kg/m^2^), **mean ± SD**	28.3 ± 6.42
Tumor site	
Breast	156 (13)
Gastrointestinal tract	173 (15)
Lung	103 (9)
Hematologic neoplasms	231 (19)
Melanoma	68 (5)
Prostate	141 (12)
Other	339 (28)
Tumor stage	
I	335 (37)
II	226 (25)
III	197 (22)
IV	144 (16)
Rural-Urban Status	
Rural	386 (26)
Urban	1,064 (73)
Employment status	
Employed full-time	471 (35)
Employed part-time	108 (8)
Not currently employed^a^	782 (57)
Had a COVID infection	
Yes	99 (7)
No	1,255 (93)
**Changes in exercise habits**	
Exercise less	439 (32)
No change in exercise habits	770 (57)
Exercise more	152 (11)

NOTE: Data might not add to 100% because of rounding. Missing values due to skip patterns or non-response not shown (race: n = 28; ethnicity: n = 99; tumor site: n = 150; tumor stage: n = 459; COVID infection: n = 7);

* N(%) if not otherwise stated;

a includes patients who are retired.

**Table 2:** Demographic and clinical characteristics by changes in exercise habits (n = 1,361).

Characteristics	Exercising less (n = 439)*	No change in exercise habits (n = 770)*	Exercising more (n = 152)*	*p*-value^a^	*p-value* ^b^
Age					
mean ± SD (range)	60 ± 15 (21–92)	62 ± 13 (23–92)	55 ± 14 (20–86)	< 0.0001	0.0001
Sex, n (%)					
Male	194 (44)	388 (50)	55 (36)	0.002	0.08
Female	245 (56)	382 (50)	97 (64)		
Race, n (%)					
White	418 (97)	735 (97)	145 (98)	0.18	0.07
Asian	6 (1)	6 (1)	0 (0)		
American Indian or Alaska Native	1 (0)	8 (1)	2 (2)		
Other	7 (2)	5 (1)	0 (0)		
Ethnicity, n (%)					
Hispanic/Latino	14 (3)	30 (4)	5 (4)	0.24	0.28
Non-Hispanic/Latino	403 (97)	676 (96)	134 (96)		
Employment status, n (%)					
Employed full-time	130 (29)	268 (35)	73 (48)	< 0.001	< 0.0001
Employed part-time	35 (8)	59 (8)	14 (9)		
Not currently employed	274 (63)	443 (58)	65 (42)		
Urban-Rural Status, n (%)					
Rural	93 (21)	243 (32)	28 (19)	< 0.0001	0.47
Urban	346 (79)	527 (68)	124 (81)		
Tumor site, n (%)					
Breast	63 (17)	74 (11)	19 (16)	0.002	0.06
Gastrointestinal tract	51 (13)	116 (16)	6 (5)		
Lung	34 (9)	53 (8)	16 (12)		
Hematologic neoplasms	73 (5)	133 (19)	25 (20)		
Melanoma	19 (18)	43 (7)	6 (5)		
Prostate	39 (10)	90 (13)	12 (10)		
Other	113 (28)	186 (26)	40 (32)		
Tumor stage, n (%)					
I	120 (41)	176 (33)	39 (42)	0.007	0.46
II	68 (23)	142 (26)	16 (17)		
III	49 (17)	132 (24)	16 (17)		
IV	47 (16)	76 (15)	21 (23)		
Current cancer patient status, n (%)					
Have cancer and currently receiving treatment	124 (30)	198 (27)	29 (21)	0.05	0.02
Have cancer and completed cancer treatment	185 (45)	319 (43)	56 (40)		
Have cancer and came for 2nd opinion	6 (1)	8 (1)	2 (1)		
Other	98 (24)	211 (29)	53 (38)		
Had a COVID infection, n (%)					
Yes	30 (7)	63 (8)	6 (4)	0.21	0.42
No	405 (93)	705 (92)	145 (96)		
Self-reported health status, n (%)					
Excellent	24 (6)	94 (13)	33 (23)	< 0.0001	< 0.0001
Very good	137 (32)	310 (40)	64 (42)		
Good	184 (41)	256 (34)	40 (26)		
Fair	79 (18)	92 (12)	13 (8)		
Poor	14 (3)	17 (2)	2 (1)		
BMI (kg/m^2^)					
mean ± SD	28.9 ± 6.60	28.1 ± 6.34	28.1 ± 6.18	0.08	0.19

NOTE: Data might not add to 100% because of rounding.

Missing values due to skip patterns or non-response not shown (race: n = 28; ethnicity: n = 99; tumor site: n = 150; tumor stage: n = 459; COVID infection: n = 7; current cancer patient status: n = 72);

* N(%) if not otherwise stated;

a comparing 3 groups of changes in exercise habits;

b comparing 2 groups of changes in exercise habits (exercise less vs. exercise more).

**Table 3:** Health behaviors by changes in exercise habits

Health behaviors	Exercising less (N = 439)	No change in exercise habits (N = 770)	Exercising more (N = 152)	*p-*value^a^	*p-value* ^b^
Current smoking status, n (%)					
Never	331 (77)	528 (69)	116 (77)	0.19	0.92
Former	94 (21)	209 (28)	31 (20)		
Current	9 (2)	24 (3)	4 (3)		
Alcohol consumption in past year, n (%)					
Never	203 (51)	365 (54)	87 (59)	< 0.001	0.001
Less than once a month	55 (14)	74 (11)	9 (6)		
Once a month to twice a week	68 (17)	127 (19)	36 (25)		
3–4 times a week to every day	73 (18)	110 (16)	14 (10)		
Change in alcohol consumption habits since COVID-19 pandemic, n (%)					
No	323 (86)	590 (92)	117 (87)	0.03	0.35
Yes, increased drinking	27 (7)	25 (4)	5 (4)		
Yes, decreased drinking	25 (7)	29 (4)	12 (9)		

NOTE: Data might not add to 100% because of rounding.

Missing values due to skip patterns or non-response not shown (current smoking status: n = 15; alcohol consumption in the past year: n = 140; change in alcohol consumption habits since COVID pandemic: n = 208);

a comparing 3 groups of changes in exercise habits;

b comparing 2 groups of changes in exercise habits (exercise less vs. exercise more).

**Table 4:** Psychosocial factors by changes in exercise habits

Psychosocial factors	Exercise less (n = 439)	No change in exercise habits (n = 770)	Exercise more (n = 152)	*p-*value^a^	*p-*value^b^
Change in daily life due to pandemic, n (%)					
Not at all	4 (1)	49 (6)	4 (3)	< 0.0001	0.14
A little bit	31 (7)	151 (20)	17 (11)		
Somewhat	82 (19)	190 (25)	25 (16)		
A moderate amount	128 (29)	207 (27)	48 (32)		
A lot	194 (44)	172 (22)	58 (38)		
Change in social interaction in past month, n (%)					
I have less social interaction	350 (79)	488 (64)	98 (65)	< 0.0001	0.0002
My social interaction has not changed much	83 (18)	244 (31)	34 (23)		
I have a more social interaction	15 (3)	38 (5)	19 (13)		
Felt lonely in past month					
Never	104 (24)	306 (40)	45 (29)	< 0.0001	0.48
Rarely	143 (33)	254 (33)	47 (31)		
Sometimes	154 (34)	172 (22)	47 (32)		
Usually	29 (7)	32 (4)	12 (7)		
Always	9 (2)	5 (1)	1 (1)		
Difficulties piling up that could not be overcome in past month, n (%)					
Never	142 (32)	391 (51)	52 (34)	< 0.0001	0.06
Almost never	140 (32)	208 (27)	61 (41)		
Sometimes	120 (27)	118 (14)	27 (17)		
Fairly often	27 (6)	34 (4)	11 (7)		
Often	10 (3)	17 (2)	1 (1)		
Financially stressed in past month, n (%)					
Not at all	180 (41)	442 (58)	64 (42)	< 0.0001	0.42
A little bit	144 (33)	188 (24)	58 (38)		
Somewhat	54 (13)	63 (8)	16 (11)		
Quite a bit	37 (8)	50 (6)	11 (7)		
Very much	23 (5)	27 (4)	3 (2)		

NOTE: Data might not add to 100% because of rounding.

Missing values due to skip patterns or non-response not shown (change in daily life due to pandemic: n = 1; change in social interaction in past month: n = 2; felt lonely in past month: n = 1; difficulties piling up that could not be overcome in past month: n = 2; financially stressed in past month: n = 1);

a comparing 3 groups of changes in exercise habits;

b comparing 2 groups of changes in exercise habits (exercise less vs. exercise more).

## Data Availability

Data can be made available upon reasonable request.
